# Gastrointestinal perforation: ultrasonographic diagnosis

**DOI:** 10.1186/2036-7902-5-S1-S4

**Published:** 2013-07-15

**Authors:** FF Coppolino, G Gatta, G Di Grezia, A Reginelli, F Iacobellis, G Vallone, M Giganti, EA Genovese

**Affiliations:** 1 University of Palermo, Department of Radiology, Palermo, Italy; 2 Second University of Naples, Department of Clinical and Experimental Internistic F. Magrassi – A. Lanzara, Naples, Italy; 3 University of Naples Federico II, Department of Biomorphological and Functional Sciences, Naples, Italy; 4 University of Ferrara, Dipartimento di Scienze Chirurgiche, Ferrara, Italy; 5 University of Cagliari, Department of Radiology, Cagliari, Italy

## Abstract

Gastrointestinal tract perforations can occur for various causes such as peptic ulcer, inflammatory disease, blunt or penetrating trauma, iatrogenic factors, foreign body or a neoplasm that require an early recognition and, often, a surgical treatment.

Ultrasonography could be useful as an initial diagnostic test to determine, in various cases the presence and, sometimes, the cause of the pneumoperitoneum.

The main sonographic sign of perforation is free intraperitoneal air, resulting in an increased echogenicity of a peritoneal stripe associated with multiple reflection artifacts and characteristic comet-tail appearance.

It is best detected using linear probes in the right upper quadrant between the anterior abdominal wall, in the prehepatic space.

Direct sign of perforation may be detectable, particularly if they are associated with other sonographic abnormalities, called indirect signs, like thickened bowel loop and air bubbles in ascitic fluid or in a localized fluid collection, bowel or gallbladder thickened wall associated with decreased bowel motility or ileus.

Neverthless, this exam has its own pitfalls. It is strongly operator-dependant; some machines have low-quality images that may not able to detect intraperitoneal free air; furthermore, some patients may be less cooperative to allow for scanning of different regions; sonography is also difficult in obese patients and with those having subcutaneous emphysema. Although CT has more accuracy in the detection of the site of perforation, ultrasound may be particularly useful also in patient groups where radiation burden should be limited notably children and pregnant women.

## Background

Gastrointestinal perforation is one of the most common cause of intraperitoneal free air; its detection is important for diagnosis of life-threatening conditions in patients with acute abdomen.

Gastrointestinal tract perforations can occur for various causes (peptic ulcer, inflammatory disease, blunt or penetrating trauma, iatrogenic factors, foreign body or a neoplasm); most of these perforations are emergency conditions requiring an early recognition and a timely surgical treatment.

The mainstay of treatment for bowel perforation is surgery.

Endoscopic, laparoscopic and laparoscopic- assisted procedures are now being increasingly performed instead of conventional laparotomy.

Moreover, if any signs and symptoms of generalized peritonitis are absent and the perforation site has sealed spontaneously, then a perforated duodenal ulcer can be treated with non-surgical procedures.

It is important to identify location and cause of the perforation correctly for appropriate management and surgical planning.

The clinical diagnosis of the site of gastrointestinal tract perforation is difficult as the symptoms may be non-specific.

## Subjects And methods

A MEDLINE and PubMed search was performed for journals before March 2013 with MeSH major terms ‘ultrasonography and ‘perforation’. Non-English speaking literature was excluded.

## Results

### Radiological anatomy

Upper and lower gastrointestinal perforation can be differentiate by transverse mesocolon such as the peritoneal cavity, usually divided into supra- and inframesocolic compartments.

Subsequently, stomach or duodenal perforation would result in supramesocolic compartment gas and distal small and large bowel perforation in inframesocolic compartment gas.

Sections of the GI tract, such as stomach, first part of duodenum (5 cm), jejunum, ileum, caecum, appendix, transverse colon, sigmoid colon and upper third rectum are found within the peritoneal cavity, and are usually mobile[1,2]. The second and third parts of the duodenum, ascending and descending colon and middle third of rectum are retroperitoneal and fixed; therefore, they may present with gas within the retroperitoneal compartment, usually the anterior pararenal space[3,4].

### Radiological free gas signs

The presence of free intraperitoneal gas on a routine radiograph usually indicates bowel perforation. Experimental studies have shown that as little as 1 ml of gas can be detected below the right hemidiaphragm on properly exposed erect chest radiographs.

Various radiological descriptions are used for specific distribution of free intraperitoneal gas, such as the Rigler sign (gas outlining both sides of the bowel), football sign (oval shaped peritoneal gas), increased lucency in the right upper quadrant (gas accumulating anterior to the liver) and triangle sign (triangular gas pocket between three loops of bowel).

Otherwise, the most relevant signs on CT are the “ligamentum teres sign” (free gas outlining the intrahepatic fissure and ligamentum teres, often due to perforation of the duodenal bulb or stomach), the “periportal free gas sign” (strongly suggests upper GI tract perforation) and the “falciform ligament sign” (free gas or a gas-fluid level crossing the mid-line and accentuating the falciform ligament, characteristic of perforation of the proximal GI tract.

Although conventional radiography is a common method for detecting small amount of intraperitoneal free air [5], imagers may not detect pneumoperitoneum or retroperitoneum in up to 49% of patients [6]; in addition, many patients with acute abdominal pain cannot stand to have a chest radiograph, so decubitus abdominal x-ray is usually used [7].

Other modalities include ultrasound, often considered an extension of clinical examination; it is routinely used to examin patients with undiagnosed abdominal pain, including those with occult gastrointestinal perforation for which the diagnosis was not previously suspected [8], despite the difficult differentiation between intraperitoneal free air and intraluminal bowel gas due to multiple reflection artifacts and dirty shadowing. Ultrasound may be particularly useful also in patient groups where radiation burden should be limited notably children and pregnant women.

### Abdominal pain patients in emergency department

Although the common causes of acute abdominal pain are acute appendicitis, diverticulitis, cholecystits and bowel obstruction, less frequent conditions may cause acute abdominal pain including perforated viscus (about 1%) and bowel ischemia.

Perforation of a peptic ulcer is now less frequent because of the availability of adequate medical therapy for peptic ulcer disease. Only 1-2% of patients have free perforation due to acute diverticulitis, also because most perforated diverticula are contained perforations.

In the emergency department, an accurate diagnosis can be made exclusively on the basis of medical history, physical examination and laboratory test findings in only a small proportion of patients.

The clinical manifestations of the various causes of acute abdominal pain usually are not straightforward; besides the variable symptoms of the underlying mechanism, a rigid abdomen usually is present [9,10].

For proper treatment, a diagnostic work-up that enables the clinician to differentiate between the various causes of acute abdominal pain is important, and ultrasonography plays an important role in this process. It is widely available and is easily accessible in the emergency department, is a real-time dynamic examination that can reveal the presence or absence of peristalsis and depict blood flow. Otherwise, the major advantage of CT, as compared with radiography and US, is that it can correctly depict the actual site of perforation in 86% of cases. Despite of the difficulty in the detection of perforation at ultrasonography,it could be diagnosed in supine patients, adiacent to the abdominal wall, the radiologist identifies echogenic lines or spots with comet-tail reverberation artifacts [11,12].

### Gastrointestinal perforation at ultrasonography

Some authors demonstrated that US has lower sensitivity than radiography (76% vs. 92%, respectively) [13] and should be used in selected cases only (clinical conditions preventing radiographs from being performed correctly, persisting clinical souspicious with negative or questionable radiographics findings, the exclusion of other acute abdominal conditions, and finally the presence of pneumoperitoneum in the patients referred for different clinical reasons) [13].

However, in literature some authors demonstrated that ultrasonography has greater accuracy (90% vs. 77%) if compared with x-ray (sensitivity 93%vs. 79%) and that ultrasonography is a useful diagnostic modality when x-rays does not reveal pneumoperitoneum in patients with suspected perforation [14,15].

Moreover, some authors demonstrate that sonography may be useful to determine not only the presence, but the cause of the pneumoperitoneum too [5]*.*

Neverthless, its detection is difficult even for an experienced sonographer [16] especially because the presence of intraperitoneal air outside the intestinal lumen is unusual and can be mistaken for air whithin the bowel.

**Figure 1:**
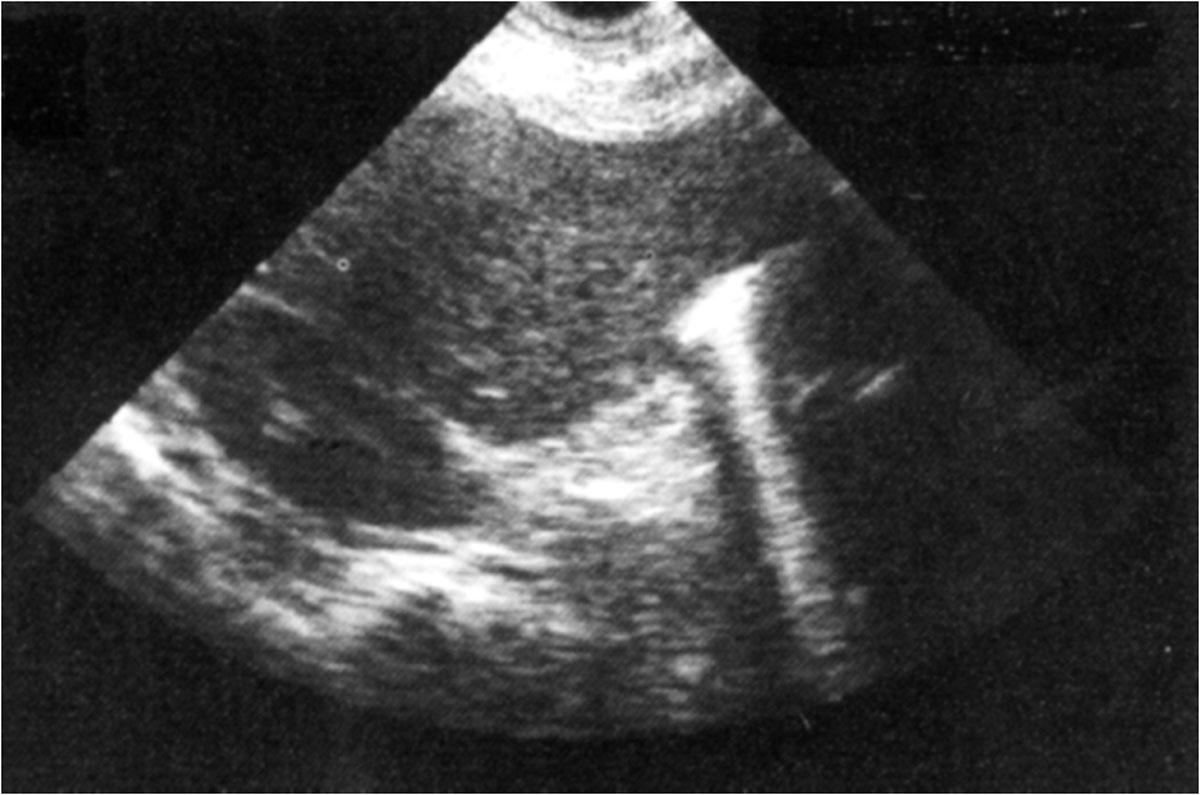
The sonographic appearance of free intraperitoneal air results form scattering of the ultrasound waves at the interface of soft tissue and air which is accompanied by reverberation of the waves between the transducer and the air

The sonographic appearance of free intraperitoneal air results form scattering of the ultrasound waves at the interface of soft tissue and air which is accompanied by reverberation of the waves between the transducer and the air (Figure 1).

This results in an increased echogenicity of a peritoneal stripe associated with multiple reflection artifacts and characteristic comet-tail appearance that can be changed by changing the patient’s position.

Conversely, intraluminal bowel gas is always associated with a more superficial, normal thin peritoneal strip.

In small air collections reverberation artifacts may not be seen, whereas in extensive pneumoperitoneum found pronounced pre-hepatic echoes with sound shadow phenomenon may obscure the underlying abdominal organs [17].

**Figure 2:**
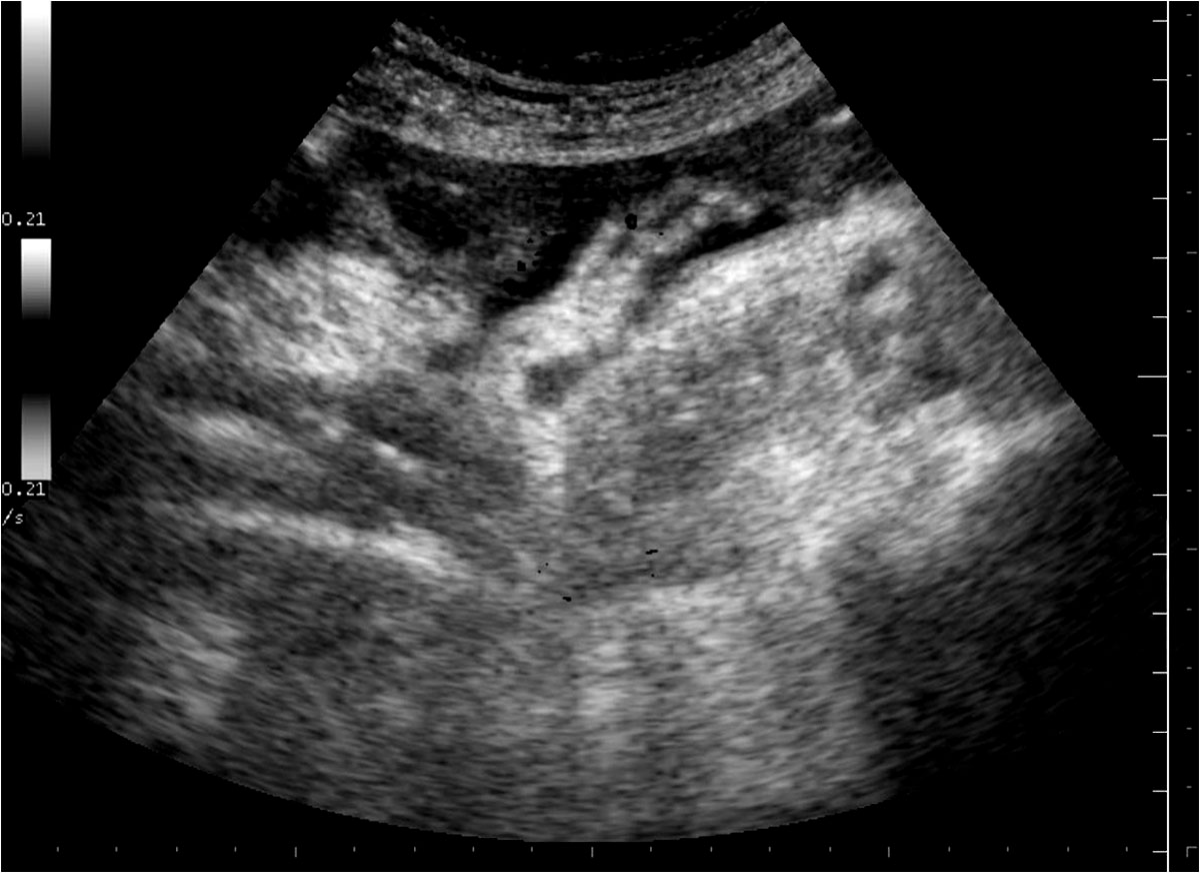
Direct sign, such localized gas collections related to bowel perforations, may be detectable, particularly if they are associated with other sonographic abnormalities, called indirect signs (thickened bowel loop and air bubbles in ascitic fluid or in a localized fluid collection, bowel or gallbladder thickened wall associated with decreased bowel motility or ileus)

Direct sign, such localized gas collections related to bowel perforations, may be detectable, particularly if they are associated with other sonographic abnormalities, called indirect signs (thickened bowel loop and air bubbles in ascitic fluid or in a localized fluid collection, bowel or gallbladder thickened wall associated with decreased bowel motility or ileus) (Figure 2) [18].

The linear array transducers (10-12MHz) are more sensitive than standard curvilinear abdominal transducers (2-5MHz) for detecting intraperitoneal free air because of the broader near-filed size and because of superior resolution in the near filed where the air usually accumulates. Table 1

**Table 1:** Direct and indirect signs of gastrointestinal perforation at Ultrasonography

DIRECT SIGNS	*Pneumoperitoneum*	• Increased echogenicity of peritoneal stripe • Step between air in costophrenic sinus and abdominal gas reflex
	
	*Pneumoretroperitoneum*	• Air around duodenum and the head of the pancreas • Vanishing vessels • Renal rind sign
**INDIRECT SIGNS**		• Intraperitoneal free fluid • Air bubbles in ascitic fluid • Thickened bowel loop • Bowel or gallbladder thickened wall with ileus

Patient should be first scanned in the supine position concentrating on the midline and right upper quadrant, then in the left lateral decubitus and prone position [5,12], although it seems impractical for uncooperative, distressed patients or acutely ill patients, who often have an ileus [8]*.*

Some authors affirm that the best position for ultrasound examination of the abdomen is supine with the thorax slightly elevated (10-20 degrees) and that the optimal prone position is in the right paramedian epigastric area in the longitudinal direction [19].

Intraperitoneal free air is best detected in the right upper quadrant between the anterior abdominal wall, in the prehepatic space; the presence of air causing an enhancement of the peritoneal stripe and moving when the patient position changes, especially in abnormal sites such as along with the fissure of ligamentum teres, should raise the suspicion of intraperitoneal free air, meanwhile intraluminal gas can be seen inside a bowel loop having a visible peristalsis and a normal wall thickness [20].

The possibility to observe motion in realtime sonography repeatedly proved to be decisive for the certain diagnosis of free air (the shifting air under patient movement and the immobility of the gas reflex under respiration).

An observed step between the air in the costophrenic sinus and the abdominal gas reflex is considered to be an additional sonographic sign [19].

In the right upper quadrant sonograms made during inspiration and expiration help to differentiate pneumoperitoneum from the adjacent lung because pneumoperitoneum overlaps the lung during inspiration, but the lung and pneumoperitoneum are separate during expiration.

In case of pneumoretroperitoneum caused by a retroperitoneal perforation it is possible to detect also air around the duodenum and the head of the pancreas and especially ventral to the great abdominal vessel which can lead to the picture of “vanishing” vessels [20,21].

Karahan introduced a new method for the detection of intraperitoneal free air, the SCISSOR MANEUVER. It consists in applying and then releasing slight pressure onto the abdominal wall with the caudal part of a parasagittally oriented linear-array probe; this maneuver could be a useful adjunct for improving the diagnostic yield of sonography [22,23].

**Figure 3:**
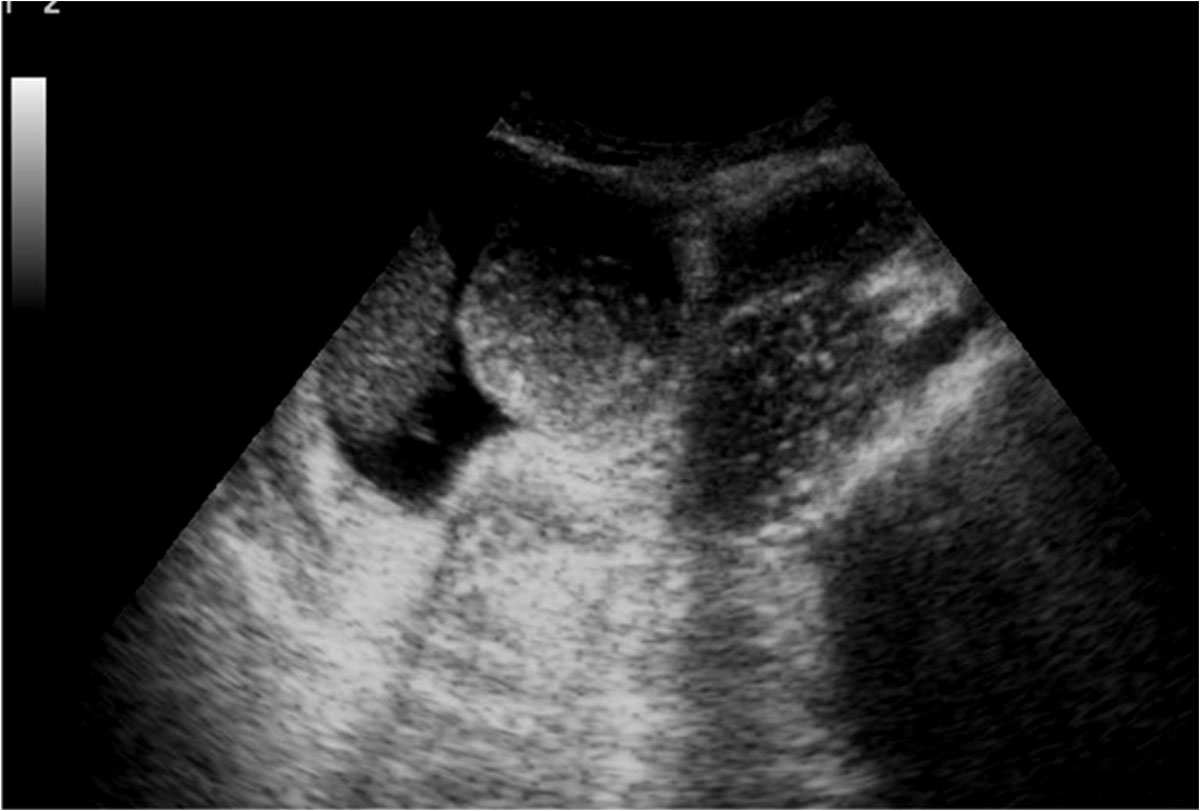
Meticulous examination focused on the patient problem may yield a causative diagnosis of peritonitis due to perforated gastric or duodenal ulcer, perforated appendicitis o diverticulitis, suggested on the basis of wall thickening, fluid accumulation, inflammatory mass, thickening of the gallbladder, hyperechogenicity of the right anterior extrarenal tissue (renal rind sign) and free intraperitoneal gas confined to the fissure for ligamentum teres

Meticulous examination focused on the patient problem may yield a causative diagnosis of peritonitis due to perforated gastric or duodenal ulcer, perforated appendicitis o diverticulitis, suggested on the basis of wall thickening, fluid accumulation, inflammatory mass ,thickening of the gallbladder [11], hyperechogenicity of the right anterior extrarenal tissue (renal rind sign) [24,25] and free intraperitoneal gas confined to the fissure for ligamentum teres (Figure 3) [23].

Gastroduodenal perforations may be suspected in patients with history of ulceration, who present with acute pain and abdominal wall rigidity, but radiological findings in these cases may be unable to confirm a clinical diagnosis.

**Figure 4:**
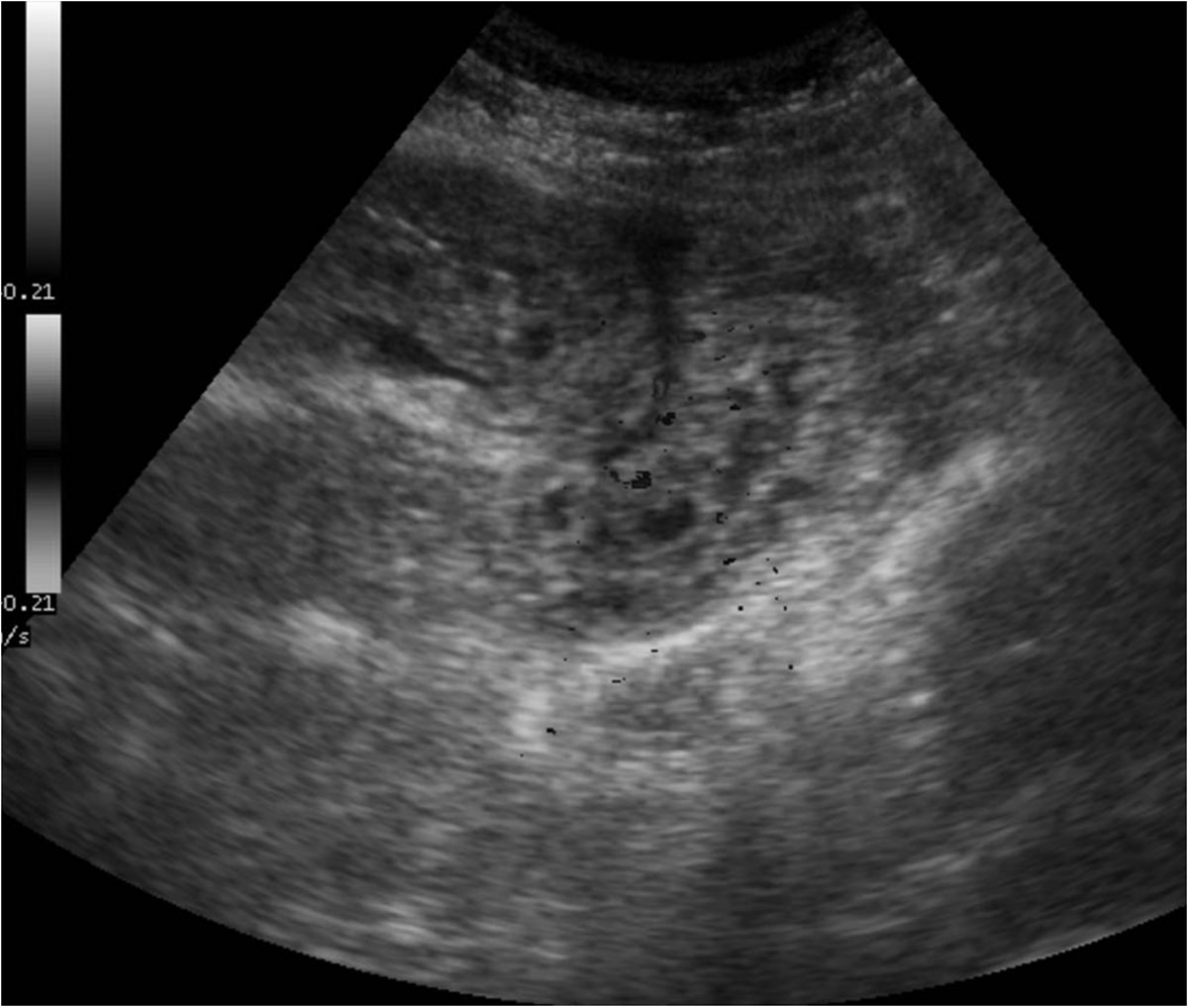
Intraperitoneal free fluid and/or reduced intestinal peristalsis at sonographic examination are considered indirect signs of gastroduodenal perforation

Intraperitoneal free fluid and/or reduced intestinal peristalsis at sonographic examination are considered indirect signs of gastroduodenal perforation (Figure 4). Ultrasonography could help to confirm intestinal paresis and the evidence of intraperitoneal free fluid [26].

Ultrasound can also detect a hypoechoic irregular lesion continuous with the jejunum suggestive of the presence of diverticula; the presence of peridiverticular hyperechoic fat, associated with US signs of extraluminal air evoked the diagnosis of a proximal jejunal diverticulitis [27]; lymph node metastasis may be seen in perforated tumors of the gastrointestinal tracts [28].

Sonography is able also to detect primary ascaridial perforation as two pairs of parallel lines, representing the worm outer margis, flanking a central sonolucent line, representing its digestive tract. It could be found also in the peritoneal cavity and in some loops of the small bowel [29].

The exam could be useful also in neonates because the sonographic findings of ascites and intraperitoneal fluid-debris levels in patients with suspected necrotizing colitis are suggestive of perforation [30]*.*

Neverthless, this exam has its own pitfalls. It is strongly operator-dependant ; some ultrasound machines have low-quality images that may not able to detect intraperitoneal free air.; furthermore, some patients may be less cooperative to allow for scanning of different regions; sonography is also difficult in obese patients and with those having subcutaneous emphysema [10,31].

### Conclusions

Ultrasound could be useful as an initial diagnostic test and CT may be reserved for patients with nondiagnostic ultrasonography results.

In conclusion, in the absence of direct or indirect findings of pneumoperitoneum, US examination is not so useful for detecting free gas, but could help to confirm intestinal paresis and intraperitoneal free fluid [31].

If perforation is suspected, patients are usually subjected to abdominal MSCT, especially because ultrasonography is operator-dependent, some patients are less cooperative, the exam is diffucult in obese patients and in those with subcutaneous emphysema; otherwise MSCT, expecially after six hours after symptoms begin, is useful to assess gastrointestinal perforation as it allows detection of even small amounts of free air in the abdomen [32]*.*

## Competing interests

The authors declare that they have no competing interests.

## Declarations

This article has been published as part of *Critical Ultrasound Journal* Volume 5 Supplement 1, 2013: Topics in emergency abdominal ultrasonography. The full contents of the supplement are available online at http://www.criticalultrasoundjournal.com/supplements/5/S1. Publication of this supplement has been funded by the University of Molise, University of Siena, University of Cagliari, University of Ferrara and University of Turin.
